# Hashimoto Thyroiditis Modifies the Association Between Nuclear Atypia and Malignancy in Bethesda III Thyroid Nodules

**DOI:** 10.3390/diagnostics16193235

**Published:** 2026-10-07

**Authors:** Adem Şentürk, Yusuf Öztürk, Emre Gönüllü, İsmail Özer, Kübra Tuba Çayir, Ahmet Tarık Harmantepe, Yavuz Selim Kahraman, Fuldem Mutlu

**Affiliations:** 1 Department of Surgical Oncology, Sakarya Training and Research Hospital, Sakarya 54100, Turkey; 2 Department of Endocrinology, Sakarya Training and Research Hospital, Sakarya 54100, Turkey; yusuf_ozturk40@hotmail.com; 3 Department of Gastroenterology Surgery, Sakarya Training and Research Hospital, Sakarya 54100, Turkey; emregonullu@gmail.com; 4 Department of Radiology, Sakarya University, Sakarya 54050, Turkey; ozer815@gmail.com (İ.Ö.); fuldem@sakarya.edu.tr (F.M.); 5 Department of İnternal Medicine, Sakarya University, Sakarya 54050, Turkey; dr.ktcayir@gmail.com; 6 Department of Gastroenterology Surgery, Van Training and Research Hospital, Van 65300, Turkey; tarikharmantepe@gmail.com; 7 Department of Surgical Oncology, Kastamonu Training and Research Hospital, Kastamonu 37150, Turkey; dr.yavuzselim06@gmail.com

**Keywords:** Bethesda III, Hashimoto thyroiditis, nuclear atypia, thyroid nodule, malignancy, interaction

## Abstract

**Background:** Nuclear atypia is associated with an increased risk of malignancy in Bethesda III thyroid nodules; however, reactive nuclear changes related to Hashimoto thyroiditis (HT) may complicate its interpretation. We investigated whether HT modifies the association between nuclear atypia and malignancy in Bethesda III nodules. **Methods:** This retrospective single-center study included 426 surgically selected patients with Bethesda III nodules who underwent thyroidectomy between January 2015 and August 2026. HT was defined by postoperative histopathology. The association between nuclear atypia and malignancy was evaluated according to HT status. Effect modification was assessed using an HT × nuclear atypia interaction term in unadjusted and multivariable logistic regression models. **Results:** HT was present in 199 (46.7%) patients. Malignancy was less frequent in patients with HT than in those without HT (26.1% vs. 38.8%, *p* = 0.006), whereas nuclear atypia was more frequent (91.0% vs. 45.8%, *p* < 0.001). In patients without HT, nuclear atypia was strongly associated with malignancy and remained an independent predictor after multivariable adjustment (adjusted OR 13.24, 95% CI 5.46–32.10; *p* < 0.001). In patients with HT, nuclear atypia was not associated with malignancy (OR 0.68, 95% CI 0.24–1.94; *p* = 0.466). The HT × nuclear atypia interaction was significant in both unadjusted (OR 0.040, *p* < 0.001) and adjusted models (OR 0.031, *p* < 0.001). **Conclusions:** HT significantly modified the association between nuclear atypia and malignancy in Bethesda III nodules. Nuclear atypia strongly predicted malignancy in the absence of HT but not in its presence. Given the postoperative definition of HT, prospective validation using preoperative HT indicators is required before clinical application.

## 1. Introduction

Thyroid nodules are among the most common endocrine disorders in adults, and their detection has increased substantially with the widespread use of high-resolution ultrasonography [1]. Although the vast majority of these nodules are benign, accurate assessment of malignancy risk is essential for selecting the most appropriate management strategy. Fine-needle aspiration (FNA) remains the most reliable diagnostic tool for evaluating the malignant potential of thyroid nodules, and cytological findings are classified according to the Bethesda System for Reporting Thyroid Cytopathology [2]. This standardized reporting system provides an estimated risk of malignancy for each diagnostic category and serves as the cornerstone of clinical decision-making.

Bethesda category III, designated as atypia of undetermined significance (AUS) in the 2023 Bethesda System for Reporting Thyroid Cytopathology, represents a heterogeneous indeterminate cytological category with a mean risk of malignancy of 22% (expected range, 13–30%) [3]. The estimated risk of malignancy within this category varies considerably, making patient management particularly challenging. Guidelines from the American Thyroid Association (ATA) and European Thyroid Association (ETA) recommend an individualized approach to Bethesda III nodules, incorporating clinical and ultrasonographic risk factors, patient preferences, repeat FNA, molecular testing where available, and, when appropriate, surveillance or surgical management [3,4,5]. Importantly, nodules exhibiting nuclear atypia suggestive of papillary thyroid carcinoma carry a substantially higher risk of malignancy than those characterized predominantly by architectural atypia, establishing nuclear atypia as one of the most influential cytological features in surgical decision-making [6].

Hashimoto’s thyroiditis is the most common autoimmune thyroid disorder and is characterized by chronic lymphocytic inflammation of the thyroid gland [7]. Persistent inflammation induces reactive changes in the follicular epithelium, including nuclear enlargement, chromatin clearing, nuclear irregularities, and nuclear grooves, all of which may closely resemble the cytological features of papillary thyroid carcinoma [8]. Consequently, AUS diagnoses are reported more frequently in patients with Hashimoto’s thyroiditis, and distinguishing true neoplastic atypia from inflammation-related reactive changes may become particularly challenging during cytological evaluation [8].

Previous studies examining the association between Hashimoto’s thyroiditis and thyroid malignancy have yielded conflicting results [9,10]. However, whether HT alters the association between specific cytological features and malignancy within Bethesda III nodules remains insufficiently understood. This question may be particularly relevant for nuclear atypia, as inflammation-related reactive nuclear alterations in HT may mimic neoplastic atypia and thereby complicate its interpretation as a marker of malignancy [11]. Specifically, whether HT modifies the relationship between nuclear atypia and malignancy in Bethesda III nodules has not been clearly established.

Therefore, the primary aim of the present study was to determine whether histopathologically confirmed Hashimoto’s thyroiditis modifies the association between nuclear atypia and malignancy in surgically treated Bethesda III (AUS) thyroid nodules. This potential effect modification was formally evaluated by testing the interaction between HT and nuclear atypia. As a secondary objective, we compared clinical, ultrasonographic, and cytological characteristics and evaluated predictors of malignancy separately in patients with and without HT.

## 2. Materials and Methods

### 2.1. Study Design and Patient Selection

This retrospective, single-center observational study included consecutive adult patients who underwent thyroidectomy at the Department of Surgical Oncology, Sakarya University Training and Research Hospital, between January 2015 and August 2026. Eligible patients were aged ≥18 years, had a preoperative fine-needle aspiration (FNA) cytology result classified as Bethesda category III [atypia of undetermined significance (AUS)], and had a definitive postoperative histopathological diagnosis.

Patients with incomplete clinical records, unavailable preoperative ultrasonographic findings, or missing cytological or histopathological data required for the study analyses were excluded. In patients with multiple thyroid nodules, the index Bethesda III nodule was matched with the corresponding postoperative histopathological lesion using nodule size, anatomical location, and thyroid lobe. Only the index nodule that led to surgical management was included, and each patient contributed a single nodule to the analysis.

Surgical management was based on routine clinical decision-making, particularly in patients with repeated AUS cytology, taking into consideration the overall clinical assessment and patient preference.

### 2.2. Ethics Approval

This study was approved by the Sakarya University Faculty of Medicine Non-Interventional Research Ethics Committee on 2 May 2023 (Application/Decision No. 158). A subsequent amendment submitted on 8 June 2026 (No. 564) was approved by the same Ethics Committee on 16 June 2026 (No. E-43012747-050.04-596832), extending the study completion date to 20 August 2026. Accordingly, all patients included in the final analysis were within the study period authorized by the Ethics Committee.

### 2.3. Data Collection

Demographic characteristics, including age and sex; laboratory findings, including serum thyroid-stimulating hormone (TSH) and anti-thyroid peroxidase antibody (anti-TPO); preoperative ultrasonographic findings; cytological characteristics; and definitive postoperative histopathological diagnoses were retrospectively retrieved from the institutional electronic medical record system.

Anti-TPO positivity was defined according to the reference range of the institutional laboratory. Ultrasonographic findings suggestive of thyroiditis and anti-TPO positivity were recorded separately and were not used to define Hashimoto thyroiditis (HT) status in the primary analyses.

### 2.4. Fine-Needle Aspiration and Cytological Evaluation

Thyroid FNA procedures were performed under ultrasonographic guidance by radiologists or endocrinologists with at least 10 years of experience in thyroid imaging and/or thyroid nodule evaluation. Cytological specimens were processed using a liquid-based cytology technique and were evaluated as part of routine clinical practice according to the Bethesda System for Reporting Thyroid Cytopathology. Only nodules classified as Bethesda category III (AUS) were included in the study.

The presence of nuclear atypia was determined retrospectively from the original cytopathology reports available in the institutional electronic medical record system; cytological slides were not re-reviewed specifically for the present study. Nuclear atypia was based on reported papillary thyroid carcinoma-like nuclear features, including nuclear enlargement, nuclear crowding or overlapping, chromatin clearing, nuclear membrane irregularities, nuclear grooves, and intranuclear cytoplasmic pseudoinclusions. No predefined numerical threshold or specific combination of these nuclear features was retrospectively applied; classification was based on the original cytopathological interpretation. Cases reported as showing nuclear atypia were classified as nuclear atypia present, whereas the remaining Bethesda III cases were classified as nuclear atypia absent.

### 2.5. Ultrasonographic Evaluation

Preoperative thyroid ultrasonography reports were retrospectively reviewed for all included patients. Ultrasonographic examinations had been performed as part of routine clinical practice, and the original ultrasonographic reports, rather than a study-specific re-evaluation of stored images, were used for data collection.

The recorded ultrasonographic characteristics included maximum nodule diameter, composition, echogenicity, margin characteristics, and echogenic foci. Thyroid parenchymal characteristics were also recorded, including heterogeneous parenchyma and findings reported as compatible with chronic thyroiditis.

For the analyses of malignancy predictors, ultrasonographic features were converted into predefined binary variables. Solid composition was defined as a completely or almost completely solid nodule. Hypoechoic echogenicity included both hypoechoic and very hypoechoic nodules. Irregular margins included lobulated or irregular margins and the presence of extrathyroidal extension. Microcalcific echogenic foci corresponded to punctate echogenic foci. Nodule size was analyzed as a continuous variable.

### 2.6. Histopathological Evaluation and Definition of Hashimoto Thyroiditis

Definitive histopathological diagnoses were obtained retrospectively from the original thyroidectomy pathology reports. Thyroidectomy specimens were evaluated and reported as part of routine clinical practice by pathologists with at least 10 years of experience. Histopathological slides were not re-reviewed specifically for the present study.

The definitive postoperative histopathological diagnosis served as the reference standard for classification of the index nodule as benign or malignant. In patients with multiple thyroid nodules, the index Bethesda III nodule was matched to the corresponding histopathological lesion using the documented nodule size, anatomical location, and thyroid lobe in the preoperative ultrasonography/FNA records and postoperative pathology report. Noninvasive follicular thyroid neoplasm with papillary-like nuclear features (NIFTP) was classified as benign for the purposes of the binary outcome analysis.

HT status was determined exclusively on the basis of the definitive postoperative histopathology report. Patients whose pathology reports documented Hashimoto thyroiditis or chronic lymphocytic thyroiditis were classified as HT-positive, whereas those without either diagnosis were classified as HT-negative. Serological anti-TPO positivity and ultrasonographic findings compatible with thyroiditis were therefore not incorporated into the definition of histopathological HT. The histological extent or grade of thyroiditis was not systematically recorded and was therefore not evaluated in the present study.

### 2.7. Study Groups and Outcome

The primary outcome was malignancy of the index Bethesda III nodule based on the definitive postoperative histopathological diagnosis.

Patients were stratified according to the presence or absence of histopathologically confirmed HT. Demographic, laboratory, ultrasonographic, cytological, and histopathological characteristics were initially compared between the HT-positive and HT-negative groups. Within each HT stratum, clinical and ultrasonographic characteristics and nuclear atypia were subsequently compared between benign and malignant nodules.

The primary analytical objective was to determine whether histopathologically confirmed HT modified the association between nuclear atypia and malignancy. Potential effect modification was formally assessed by testing an interaction term between HT status and nuclear atypia (HT × nuclear atypia). As secondary analyses, factors associated with malignancy were evaluated separately in the HT-positive and HT-negative groups.

### 2.8. Statistical Analysis

All statistical analyses were performed using IBM SPSS Statistics for Windows, Version 22.0 (IBM Corp., Armonk, NY, USA). Firth penalized logistic regression sensitivity analyses were performed using R with the logistf package.

Continuous variables were summarized as mean ± standard deviation (SD) or median (range), as appropriate according to their distribution, whereas categorical variables were presented as frequencies and percentages. Normality of continuous variables was assessed using the Kolmogorov–Smirnov test. Comparisons between two independent groups were performed using the independent-samples *t*-test for normally distributed continuous variables and the Mann–Whitney *U* test for non-normally distributed continuous variables. Categorical variables were compared using Pearson’s chi-square test or Fisher’s exact test, as appropriate.

Binary logistic regression analyses were performed using the definitive histopathological outcome (benign vs. malignant) as the dependent variable. Odds ratios (ORs) with corresponding 95% confidence intervals (CIs) were calculated.

To formally evaluate whether HT modified the association between nuclear atypia and malignancy, an interaction term between histopathological HT status and nuclear atypia (HT × nuclear atypia) was constructed. The interaction was first evaluated in an unadjusted logistic regression model containing the main effects of HT and nuclear atypia together with their interaction term.

A multivariable interaction model was subsequently constructed to determine whether the interaction persisted after adjustment for potential confounding and malignancy-associated characteristics. The adjusted model included HT status, nuclear atypia, the HT × nuclear atypia interaction term, age, nodule size, solid composition, hypoechoic echogenicity, irregular margins, and microcalcific echogenic foci.

The incremental contribution of the interaction term was further evaluated using hierarchical logistic regression. In the first block, the model included the main effects of HT and nuclear atypia together with age, nodule size, solid composition, hypoechoic echogenicity, irregular margins, and microcalcific echogenic foci. In the second block, the HT × nuclear atypia interaction term was added while all other variables were retained. Improvement in model fit following addition of the interaction term was assessed by the change in the likelihood-ratio chi-square statistic (Δχ^2^). Calibration of the final model was assessed using the Hosmer–Lemeshow goodness-of-fit test, and the proportion of variation explained by the model was summarized using Nagelkerke’s R^2^. Discrimination of the final adjusted interaction model was assessed using the area under the receiver operating characteristic curve (AUC) with its 95% confidence interval.

To further characterize the direction of the observed interaction and identify malignancy-associated factors within each HT stratum, separate logistic regression analyses were performed for HT-negative and HT-positive patients. Univariable logistic regression analyses were first performed for individual clinical, ultrasonographic, and cytological variables. Variables with *p* < 0.10 in the univariable analyses were entered into the corresponding multivariable logistic regression models using the enter method. No additional variables were forced into the models solely on the basis of clinical relevance. Adjusted ORs with 95% CIs were reported for the multivariable models. Given the sparse distribution of microcalcific echogenic foci, Firth penalized logistic regression was additionally performed as a sensitivity analysis for the HT-stratified multivariable models. The same covariates included in the corresponding conventional multivariable logistic regression models were retained in the penalized models.

All statistical tests were two-sided, and a *p* value < 0.05 was considered statistically significant.

## 3. Results

### 3.1. Clinicopathological Characteristics According to Hashimoto Thyroiditis Status

A total of 426 patients with Bethesda III (AUS) thyroid nodules who underwent thyroidectomy were included in the study. Histopathological examination confirmed Hashimoto thyroiditis (HT) in 199 patients (46.7%), whereas 227 patients (53.3%) had no evidence of HT.

Patients with HT were more frequently female than those without HT (86.4% vs. 77.5%, *p* = 0.018) and had significantly smaller nodules (22.1 ± 14.9 mm vs. 25.9 ± 17.5 mm, *p* = 0.017). Nodule composition and echogenicity distributions also differed significantly according to HT status (*p* < 0.001 and *p* = 0.001, respectively). Nuclear atypia was substantially more frequent in the HT-positive group (91.0% vs. 45.8%, *p* < 0.001), whereas the overall malignancy rate was significantly lower than in the HT-negative group (26.1% vs. 38.8%, *p* = 0.006) (Table 1).

### 3.2. Association Between Nuclear Atypia and Malignancy According to Hashimoto Thyroiditis Status

The association between nuclear atypia and malignancy differed markedly according to HT status. Among patients without HT, nuclear atypia was present in 83.0% of malignant nodules compared with 22.3% of benign nodules (*p* < 0.001). In contrast, among patients with HT, nuclear atypia was similarly frequent in malignant and benign nodules (88.5% vs. 91.8%, *p* = 0.466).

Among HT-negative patients, malignant nodules were also more frequently solid, hypoechoic, had irregular margins, and contained microcalcific echogenic foci than benign nodules (all *p* < 0.05). In the HT-positive group, malignant nodules occurred in younger patients and were smaller and more frequently solid, hypoechoic, and associated with microcalcific echogenic foci, whereas irregular margins were not significantly associated with malignancy (Table 2).

### 3.3. Interaction Between Hashimoto Thyroiditis and Nuclear Atypia

To formally evaluate whether HT modified the association between nuclear atypia and malignancy, an interaction term between histopathological HT and nuclear atypia was incorporated into logistic regression models. In the unadjusted interaction model, the HT × nuclear atypia interaction was strongly associated with malignancy (OR = 0.040, 95% CI: 0.012–0.139; *p* < 0.001).

The interaction remained significant after adjustment for age, nodule size, solid composition, hypoechoic echogenicity, irregular margins, and microcalcific echogenic foci (adjusted OR = 0.031, 95% CI: 0.007–0.136; *p* < 0.001). Addition of the interaction term to the adjusted model resulted in a significant improvement in model fit (Δχ^2^ = 19.184, df = 1; *p* < 0.001). The final model showed adequate calibration (Hosmer–Lemeshow *p* = 0.431) and a Nagelkerke R^2^ of 0.596 (Table 3).

These findings indicate that the association between nuclear atypia and malignancy differed significantly according to the presence of histopathological HT.

### 3.4. Stratified Predictors of Malignancy According to Hashimoto Thyroiditis Status

Stratified logistic regression analyses were subsequently performed to characterize the direction of the observed interaction. In the HT-negative group, nuclear atypia was strongly associated with malignancy in univariate analysis (OR = 16.955, 95% CI: 8.553–33.610; *p* < 0.001) and remained an independent predictor after multivariable adjustment (OR = 13.241, 95% CI: 5.462–32.100; *p* < 0.001). Solid composition (OR = 4.156, 95% CI: 1.394–12.389; *p* = 0.011) and microcalcific echogenic foci (OR = 75.005, 95% CI: 9.007–624.623; *p* < 0.001) were also independently associated with malignancy (Table 4).

In contrast, nuclear atypia was not associated with malignancy in the HT-positive group in univariate analysis (OR = 0.681, 95% CI: 0.239–1.940; *p* = 0.466). In the multivariable model, younger age (OR = 0.953 per year, 95% CI: 0.918–0.988; *p* = 0.010), solid composition (OR = 2.937, 95% CI: 1.124–7.677; *p* = 0.028), hypoechoic echogenicity (OR = 5.075, 95% CI: 1.991–12.940; *p* = 0.001), and microcalcific echogenic foci (OR = 117.995, 95% CI: 12.040–1156.383; *p* < 0.001) were independently associated with malignancy (Table 5).

Given the sparse distribution of microcalcific echogenic foci, Firth penalized logistic regression was additionally performed as a sensitivity analysis using the same covariates as the corresponding conventional multivariable models. The HT-negative and HT-positive models included 88 and 52 malignancy events, corresponding to 12.6 and 10.4 events per variable, respectively. The findings were consistent with the conventional logistic regression analyses; microcalcific echogenic foci remained independently associated with malignancy in both HT strata, while nuclear atypia remained independently associated with malignancy in the HT-negative group (Table S1).

## 4. Discussion

The principal finding of the present study was that histopathologically confirmed HT significantly modified the association between nuclear atypia and malignancy in surgically treated Bethesda III (AUS) thyroid nodules. The HT × nuclear atypia interaction was significant in both the unadjusted and multivariable-adjusted models and remained significant after adjustment for age, nodule size, and major ultrasonographic risk features. Moreover, the addition of the interaction term significantly improved model fit, providing formal statistical evidence that the association between nuclear atypia and malignancy differed according to HT status. Stratified analyses further characterized the direction of this interaction: nuclear atypia was a strong independent predictor of malignancy in patients without HT, whereas no significant association was observed in patients with HT. In contrast, solid composition and microcalcific echogenic foci remained independently associated with malignancy in both groups. These findings suggest that HT may specifically modify the association between nuclear atypia and malignancy in Bethesda III nodules.

Although the association between HT and differentiated thyroid carcinoma has been extensively investigated, the nature of this relationship remains controversial, and previous studies have yielded inconsistent results [9,10]. In our cohort, the overall malignancy rate was lower among patients with HT, consistent with some previous reports. However, this finding should not be interpreted as evidence of a protective effect of HT against thyroid cancer. Because our study included only surgically treated Bethesda III nodules, factors related to cytological classification, referral for surgery, and patient selection may have contributed to the observed malignancy rates. More importantly, our findings suggest that the relevance of HT in this setting may extend beyond its association with overall malignancy risk to its modifying effect on the relationship between nuclear atypia and malignancy.

The most important and potentially novel finding of our study was the significant interaction between HT and nuclear atypia. Nuclear atypia is considered one of the most important cytological features associated with malignancy in Bethesda III nodules [12,13]. In our cohort, nuclear atypia was strongly associated with malignancy in patients without HT and remained an independent predictor after multivariable adjustment. In contrast, no significant association between nuclear atypia and malignancy was observed among patients with HT. Importantly, this difference was formally tested through an interaction analysis rather than inferred solely from statistical significance in one subgroup and its absence in the other. The HT × nuclear atypia interaction remained significant after adjustment for relevant clinical and ultrasonographic characteristics, and the addition of the interaction term significantly improved model fit. These findings therefore provide evidence that the association between nuclear atypia and malignancy in Bethesda III nodules may depend on the underlying histopathological inflammatory background.

The most plausible explanation for this finding is the chronic lymphocytic inflammation associated with HT, which can induce reactive nuclear alterations in follicular epithelial cells. Inflammation-related changes, including nuclear enlargement, chromatin clearing, nuclear irregularities, and nuclear grooves, may closely resemble the characteristic nuclear features of papillary thyroid carcinoma [14,15,16]. Consequently, a proportion of nuclear atypia observed in patients with HT may represent reactive inflammatory changes rather than true neoplastic atypia. Indeed, nuclear atypia was present in 91.0% of patients with HT compared with 45.8% of those without HT in our cohort, despite the lower overall malignancy rate in the HT group. Furthermore, among patients with HT, the frequency of nuclear atypia did not differ significantly between benign and malignant nodules. These observations support the hypothesis that inflammation-related reactive changes may weaken the association between cytologically reported nuclear atypia and true malignancy in the presence of HT. However, because of the retrospective nature of our study, this mechanism cannot be directly established and should be regarded as a biologically plausible but unproven hypothesis. Direct evidence from studies incorporating standardized centralized cytopathological review and molecular characterization is needed to determine whether HT-related reactive nuclear changes account for the attenuated association between nuclear atypia and malignancy observed in HT-positive nodules.

Unlike nuclear atypia, some established suspicious ultrasonographic features remained independently associated with malignancy across HT strata. Solid composition and microcalcific echogenic foci were independently associated with malignancy in both patients with and without HT. Hypoechogenicity, however, was independently associated with malignancy only in patients with HT. Irregular margins were associated with malignancy in unadjusted analyses among patients without HT but did not retain independent significance in the multivariable model. These findings are consistent with the clinical relevance of ultrasonographic features associated with a high risk of malignancy in current ATA and ETA guidelines [4,5]. However, because our study was not designed to directly compare diagnostic performance measures such as sensitivity, specificity, or AUC between HT groups, it would be inappropriate to conclude that the diagnostic performance of ultrasonographic features is preserved in the presence of HT. Rather, our findings demonstrate that certain suspicious ultrasonographic characteristics remained associated with malignancy in both HT strata. This observation further emphasizes the importance of interpreting cytological findings within their ultrasonographic context, particularly in patients with HT.

From a clinical perspective, our findings suggest that nuclear atypia may not carry the same implication for malignancy in Bethesda III nodules with and without HT. Accordingly, nuclear atypia occurring in an inflammatory background associated with HT may warrant interpretation within the broader ultrasonographic and clinical context rather than in isolation. ATA and ETA guidelines for indeterminate/Bethesda III nodules emphasize integrated risk assessment and include repeat FNA and, where available and appropriate, molecular testing among the options used to refine management decisions [4,5]. If prospectively validated using preoperative indicators of HT, our findings raise the possibility that the inflammatory thyroid background could provide additional context when interpreting nuclear atypia within this decision-making process. An important consideration, however, is that HT in the present study was defined on the basis of postoperative histopathology and was therefore not available as a preoperative classifier at the time of surgical decision-making. Consequently, our findings should not be interpreted as supporting changes to current thresholds for surgery or established management algorithms. Prospective studies using preoperatively available indicators of HT are needed to determine whether the observed effect modification can be reproduced in a clinically applicable setting.

Several limitations should be acknowledged. The retrospective, single-center design introduces the potential for selection bias. Furthermore, because only patients who underwent thyroidectomy were included, the findings may not be fully generalizable to the broader population of patients with Bethesda III cytology. Surgical decisions were made as part of routine clinical practice, particularly in the context of repeated AUS results, overall clinical assessment, and patient preference, which may have contributed to selection and spectrum bias in this surgically selected cohort. Detailed surgical indications were not systematically available for comparison between the HT groups; therefore, we could not determine whether the factors leading to thyroidectomy differed according to HT status. Such differential selection could potentially have influenced both the observed malignancy rates and the magnitude of the HT × nuclear atypia interaction. Second, HT was defined according to postoperative histopathology. Although this provided a pathology-based classification of HT, preoperative indicators of HT were not used to define HT status, limiting the direct preoperative applicability of our findings. Third, cytological slides and ultrasonographic images were not re-reviewed specifically for this study; nuclear atypia and ultrasonographic characteristics were retrospectively derived from the original clinical reports. Thus, nuclear atypia reflects routine clinical reporting rather than a standardized centralized cytopathological assessment, and some degree of misclassification cannot be excluded. This may be particularly relevant in patients with HT, in whom reactive nuclear changes may have been classified as nuclear atypia and could have attenuated the observed association between nuclear atypia and malignancy toward the null. In addition, the number of patients with HT but without nuclear atypia was relatively small, which may have reduced the precision of estimates within this subgroup. The very large effect estimates and wide CIs observed for microcalcific echogenic foci indicate sparse data and substantial imprecision. Although the associations remained significant in the Firth penalized sensitivity analyses, the corresponding ORs remained large with wide CIs and should therefore be regarded as unstable rather than interpreted as precise estimates of the magnitude of association. Finally, molecular testing was not available; therefore, we could not assess whether the observed modification of the association between nuclear atypia and malignancy by HT was independent of, or related to, underlying molecular alterations. Accordingly, our findings require validation in larger, multicenter, prospective cohorts integrating standardized cytopathological assessment, preoperatively available indicators of HT, and molecular data to further characterize this interaction and determine its potential clinical relevance.

## 5. Conclusions

In conclusion, histopathologically confirmed HT significantly modified the association between nuclear atypia and malignancy in surgically treated Bethesda III (AUS) thyroid nodules. Nuclear atypia was a strong independent predictor of malignancy in patients without HT, whereas this association was not observed in patients with HT, and this difference was supported by a significant formal interaction. In contrast, solid composition and microcalcific echogenic foci remained independently associated with malignancy in both HT strata. These findings suggest that the implication of nuclear atypia for malignancy in Bethesda III cytology may vary according to the underlying inflammatory thyroid background and highlight the importance of interpreting cytological findings within the broader clinical and ultrasonographic context. However, given the retrospective, surgically selected cohort and the postoperative histopathological definition of HT, these findings should be considered hypothesis-generating and require prospective validation with preoperative HT indicators before incorporation into clinical risk stratification.

## Figures and Tables

**Table 1:** Baseline characteristics of patients with AUS thyroid nodules according to histopathological Hashimoto thyroiditis.

Variable	Hashimoto (−) *n* = 227	Hashimoto (+) *n* = 199	*p* Value
Demographic characteristics			
Age, years	48.2 ± 12.4	49.0 ± 12.5	0.515
Female sex, n (%)	176 (77.5)	172 (86.4)	0.018
Laboratory findings			
Anti-TPO positivity, n (%)	0 (0.0)	179 (89.9)	<0.001
Ultrasonographic characteristics			
Nodule size, mm	25.9 ± 17.5	22.1 ± 14.9	0.017
Composition, n (%)			<0.001
Cystic	39 (17.2)	12 (6.0)	
Spongiform	6 (2.6)	12 (6.0)	
Mixed cystic–solid	37 (16.3)	64 (32.2)	
Solid	145 (63.9)	111 (55.8)	
Echogenicity, n (%)			0.001
Isoechoic	63 (27.8)	64 (32.2)	
Hyperechoic	16 (7.0)	26 (13.1)	
Hypoechoic	109 (48.0)	97 (48.7)	
Anechoic	39 (17.2)	12 (6.0)	
Irregular margin, n (%)	31 (13.7)	19 (9.5)	0.189
Echogenic foci, n (%)			0.077
None/comet-tail artifact	153 (67.4)	141 (70.9)	
Macrocalcification	36 (15.9)	39 (19.6)	
Microcalcification	38 (16.7)	19 (9.5)	
Ultrasonographic thyroiditis, n (%)	0 (0.0)	166 (83.4)	<0.001
Cytological findings			
Nuclear atypia, n (%)	104 (45.8)	181 (91.0)	<0.001
Final histopathology			
Malignancy, n (%)	88 (38.8)	52 (26.1)	0.006

Values are presented as mean ± standard deviation or n (%). *p* values were calculated using the independent-samples *t*-test for continuous variables and the chi-square test or Fisher’s exact test for categorical variables, as appropriate. Anti-TPO positivity is presented for descriptive purposes only and is not used to define HT status.

**Table 2:** Comparison of benign and malignant AUS nodules according to histopathological Hashimoto thyroiditis.

Variable	Hashimoto (−) Benign (*n* = 139)	Hashimoto (−) Malignant (*n* = 88)	*p*	Hashimoto (+) Benign (*n* = 147)	Hashimoto (+) Malignant (*n* = 52)	*p*
Age (years)	49.8 ± 12.3	45.8 ± 12.1	0.019	51.0 ± 12.6	43.3 ± 10.4	<0.001
Female sex, n (%)	104 (74.8)	72 (81.8)	0.218	124 (84.4)	48 (92.3)	0.150
Nodule size (mm)	27.5 ± 17.2	23.5 ± 17.7	0.091	23.7 ± 15.0	17.8 ± 13.7	0.015
Solid component, n (%)	68 (48.9)	77 (87.5)	<0.001	70 (47.6)	41 (78.8)	<0.001
Hypoechoic echogenicity, n (%)	55 (39.6)	54 (61.4)	<0.001	57 (38.8)	40 (76.9)	<0.001
Irregular margin, n (%)	11 (7.9)	20 (22.7)	0.002	12 (8.2)	7 (13.5)	0.264
Microcalcification, n (%)	1 (0.7)	37 (42.0)	<0.001	1 (0.07)	19 (36.5)	<0.001
Nuclear atypia, n (%)	31 (22.3)	73 (83.0)	<0.001	135 (91.8)	46 (88.5)	0.466

Values are presented as mean ± standard deviation or n (%). Continuous variables were compared using the independent-samples *t*-test, whereas categorical variables were compared using the Pearson chi-square test or Fisher’s exact test, as appropriate.

**Table 3:** Interaction between histopathological Hashimoto thyroiditis and nuclear atypia for predicting malignancy in AUS thyroid nodules.

Variable	Model 1 Unadjusted OR (95% CI)	*p*	Model 2 Adjusted OR (95% CI)	*p*
Hashimoto thyroiditis	3.600 (1.176–11.022)	0.025	5.551 (1.499–20.560)	0.010
Nuclear atypia	16.955 (8.553–33.610)	<0.001	12.477 (5.155–30.200)	<0.001
Hashimoto thyroiditis × nuclear atypia	0.040 (0.012–0.139)	<0.001	0.031 (0.007–0.136)	<0.001
Age (per year)	—	—	0.963 (0.940–0.988)	0.003
Nodule size (per mm)	—	—	0.982 (0.964–1.001)	0.061
Solid component	—	—	3.186 (1.575–6.445)	0.001
Hypoechoic echogenicity	—	—	1.748 (0.964–3.168)	0.066
Irregular margin	—	—	1.558 (0.667–3.641)	0.306
Microcalcific echogenic foci	—	—	158.132 (20.507–1219.355)	<0.001

Model 1 was the unadjusted interaction model and included histopathological Hashimoto thyroiditis, nuclear atypia, and the HT × nuclear atypia interaction term. Model 2 was the adjusted interaction model and additionally included age, nodule size, solid component, hypoechoic echogenicity, irregular margin, and microcalcific echogenic foci. Addition of the Hashimoto thyroiditis × nuclear atypia interaction term to the adjusted model significantly improved model fit (Δχ^2^ = 19.184, df = 1, *p* < 0.001). The final adjusted model showed adequate calibration (Hosmer–Lemeshow *p* = 0.431), a Nagelkerke R^2^ of 0.596, and an AUC of 0.902 (95% CI, 0.873–0.932). Odds ratios (ORs) with 95% confidence intervals (CIs) are presented.

**Table 4:** Univariate and multivariate logistic regression analysis for predictors of malignancy in Hashimoto-negative AUS nodules.

Variable	Univariate OR (95% CI)	*p*	Multivariate OR (95% CI)	*p*
Age (per year)	0.974 (0.952–0.996)	0.021	0.968 (0.935–1.003)	0.072
Male sex	1.514 (0.780–2.940)	0.220	—	—
Nodule size (per mm)	0.986 (0.970–1.002)	0.093	0.981 (0.958–1.004)	0.107
Solid component	7.309 (3.579–14.925)	<0.001	4.156 (1.394–12.389)	0.011
Hypoechoic echogenicity	2.426 (1.403–4.194)	0.002	0.698 (0.292–1.669)	0.419
Irregular margin	3.422 (1.550–7.559)	0.002	2.053 (0.664–6.346)	0.212
Microcalcific echogenic foci	100.118 (13.387–748.761)	<0.001	75.005 (9.007–624.623)	<0.001
Nuclear atypia	16.955 (8.553–33.610)	<0.001	13.241 (5.462–32.100)	<0.001

Univariate and multivariate logistic regression analyses evaluating predictors of malignancy in Hashimoto thyroiditis-negative AUS thyroid nodules. Variables with *p* < 0.10 in univariate analysis were entered into the multivariate logistic regression model. Odds ratios (ORs) with 95% confidence intervals (CIs) are presented.

**Table 5:** Univariate and multivariate logistic regression analysis for predictors of malignancy in Hashimoto-positive AUS nodules.

Variable	Univariate OR (95% CI)	*p*	Multivariate OR (95% CI)	*p*
Age (per year)	0.947 (0.920–0.975)	<0.001	0.953 (0.918–0.988)	0.010
Male sex	0.449 (0.148–1.367)	0.159	—	—
Nodule size (per mm)	0.970 (0.945–0.995)	0.017	0.981 (0.949–1.014)	0.257
Solid component	4.098 (1.957–8.621)	<0.001	2.937 (1.124–7.677)	0.028
Hypoechoic echogenicity	5.263 (2.548–10.872)	<0.001	5.075 (1.991–12.940)	0.001
Irregular margin	1.750 (0.649–4.716)	0.269	—	—
Microcalcific echogenic foci	84.061 (10.864–650.407)	<0.001	117.995 (12.040–1156.383)	<0.001
Nuclear atypia	0.681 (0.239–1.940)	0.466	—	—

Univariate and multivariate logistic regression analyses evaluating predictors of malignancy in Hashimoto thyroiditis-positive AUS thyroid nodules. Variables with *p* < 0.10 in univariate analysis were entered into the multivariate logistic regression model. Odds ratios (ORs) with 95% confidence intervals (CIs) are presented.

## Data Availability

The data presented in this study are available on request from the corresponding author due to ethical and patient confidentiality restrictions.

## Supplementary Materials

The following supporting information can be downloaded at https://www.mdpi.com/article/10.3390/diagnostics16193235/s1: Table S1. Firth penalized multivariable logistic regression sensitivity analysis for predictors of malignancy in Hashimoto-negative and Hashimoto-positive AUS nodules.
