# Supplementary material for: Hashimoto Thyroiditis Modifies the Association Between Nuclear Atypia and Malignancy in Bethesda III Thyroid Nodules
Source: Diagnostics (Basel). 2026 Oct 7;16(19):3235. doi: 10.3390/diagnostics16193235 (PMC13663918; doi:10.3390/diagnostics16193235)
Supplement: Supplementary file 1 [file diagnostics-16-03235-s001.zip › diagnostics-4584216-supplementary.pdf]

Table S1. Firth penalized multivariable logistic regression sensitivity analysis for predictors of malignancy in Hashimoto-negative and Hashimoto-positive AUS nodules.

**Variable**	**Hashimoto (−) OR (95% CI)**	**P**	**Hashimoto (+) OR (95% CI)**	**P**
Age (per year)	0.970 (0.936–1.001)	0.071	0.955 (0.923–0.989)	**0.010**
Male sex	—	—	—	—
Nodule size (per mm)	0.983 (0.960–1.003)	0.101	0.982 (0.951–1.014)	0.258
Solid component	4.087 (1.401–11.440)	**0.014**	2.791 (1.051–7.100)	**0.032**
Hypoechoic echogenicity	0.704 (0.301–1.653)	0.399	4.809 (1.821–11.849)	**0.001**
Irregular margin	1.957 (0.651–5.743)	0.231	—	—
Microcalcific echogenic foci	59.144 (9.104–451.329)	**<0.001**	69.756 (10.246–602.374)	**<0.001**
Nuclear atypia	12.153 (5.101–31.005)	**<0.001**	—	—

Firth penalized multivariable logistic regression sensitivity analyses evaluating predictors of malignancy in Hashimoto thyroiditis-negative and Hashimoto thyroiditis-positive AUS thyroid nodules. The same variables included in the corresponding conventional multivariable logistic regression models were entered into the Firth penalized models. The Hashimoto-negative model included 88 malignancy events and 7 variables (12.6 events per variable), whereas the Hashimoto-positive model included 52 malignancy events and 5 variables (10.4 events per variable). Odds ratios (ORs) with 95% confidence intervals (CIs) are presented.
